# Calibrated Feature Fusion: Enhancing Few-Shot Industrial Anomaly Detection via Cross-Stage Representation Alignment

**DOI:** 10.3390/s26072164

**Published:** 2026-03-31

**Authors:** Shuangjun Zheng, Songtao Zhang, Zhihuan Huang, Kuoteng Sun, Yuzhong Gong, Jiayan Wen, Eryun Liu

**Affiliations:** 1 College of Information Science & Electronic Engineering, Zhejiang University, Hangzhou 310027, China; 22360706@zju.edu.cn (S.Z.);; 2 Liuzhou Bureau, Extra High Voltage Transmission Company, China Southern Power Grid Co., Ltd., Liuzhou 545026, China; 3 College of Control Science and Engineering, Zhejiang University, Hangzhou 310027, China; 4 School of Automation (School of Artificial Intelligence), Guangxi University of Science and Technology, Liuzhou 545006, China

**Keywords:** industrial anomaly detection, few-shot learning, cross-domain adaptation, vision transformers, feature fusion, cross-stage alignment

## Abstract

Few-shot industrial anomaly detection technology has received more and more attention because it does not require a large number of abnormal samples to train. Recent few-shot industrial anomaly detection methods commonly fuse multi-stage features from frozen vision transformers for anomaly scoring. However, we find that such direct fusion suffers from cross-stage representation misalignment—shallow and deep features differ significantly in scale and semantic granularity, leading to inconsistent anomaly maps and degraded localization. To address this problem, we propose Calibrated Feature Fusion (CFF), a lightweight adapter that enhances feature fusion via cross-stage representation alignment. The CFF module can be integrated into existing state-of-the-art frameworks and operates effectively in few-shot settings. Experiments on MVTec AD and VisA show that CFF consistently improves the state-of-the-art method across 1/2/4-shot settings, achieving gains of up to +1.6% AUROC and +4.1% AP in pixel-level segmentation. Notably, CFF enhances both precision and recall in four-shot scenarios. Ablation studies confirm that cross-stage alignment is key to stable multi-stage fusion.

## 1. Introduction

Automated visual inspection is critical for modern industrial quality control, particularly in high-stakes domains like semiconductor manufacturing and precision electronics. Even minor surface defects—scratches, dents, or contamination—can lead to product failure or costly recalls. In practical industrial scenarios, these subtle defects are often further obscured by complex imaging conditions such as uneven illumination, low contrast, and environmental interference [1], making defect localization more challenging. Traditional rule-based or template matching approaches struggle with the diversity and subtlety of real-world anomalies [2,3,4,5], motivating a shift toward data-driven deep learning methods [6,7,8,9,10,11]. However, a fundamental bottleneck remains: the extreme scarcity of labeled anomalous samples. Anomalies are by nature rare, unpredictable, and often unique to specific production batches. Collecting comprehensive defect datasets is not only labor intensive but frequently infeasible, especially when production lines rapidly switch between product models.

This challenge has driven significant interest in unsupervised and few-shot anomaly detection (FSAD), where models adapt to new object categories using only a small number (e.g., 1–4) of normal reference images, without access to anomalous examples during training [6,7]. This paradigm closely mirrors flexible manufacturing scenarios, where retraining from scratch for every new item is impractical. Compounding the difficulty is domain shift: features learned on one category (e.g., metal screws) may not generalize well to another (e.g., plastic capsules), demanding robust cross-domain generalization.

Recent advances in large-scale pretrained vision models have opened new avenues for FSAD. Vision–language models like CLIP [12] enable zero-shot anomaly detection by aligning image patches with textual prompts describing “normal” instances [13]. Self-supervised ViTs such as DINOv2 [14] learn rich hierarchical representations capturing both local texture and global structure across stages. State-of-the-art methods increasingly combine these strengths. For example, AD-DINOv3 [15] leverages multi-stage patch tokens aligned with CLIP text embeddings; April-GAN [16] projects stage-wise ViT features into the CLIP space via linear layers and fuses similarity scores against memory bank references.

A key challenge in such multi-stage frameworks lies in reconciling heterogeneous feature representations across network depths—a phenomenon widely observed in vision transformers [17,18]. Shallow layers capture fine-grained details at high resolution, producing numerous small, scattered responses sensitive to local textures. In contrast, deeper stages encode holistic semantics at lower resolution, yielding fewer but broader activation patterns corresponding to structural deviations. While this hierarchy is beneficial, the direct fusion of such divergent signals—typically through naive summation or averaging—can result in suboptimal localization due to distributional mismatches in activation statistics, dynamic ranges, and semantic emphasis.

To mitigate this, recent methods employ stage-wise projections [16] or adaptive weighting schemes [15]. However, these transformations are often fixed or lack explicit constraints that ensure consistency across stages under limited supervision. Crucially, they do not fully exploit the few available normal samples to calibrate inter-stage coherence.

In response, we propose Calibrated Feature Fusion (CFF), a lightweight, plug-and-play module that enhances multi-stage fusion by explicitly aligning feature distributions across stages. CFF introduces a minimal adapter after stage-wise projections, trained end-to-end using a symmetric similarity loss that encourages adjacent stages to exhibit consistent distributional characteristics in the shared embedding space. Importantly, CFF operates only during few-shot fine-tuning when target-domain data is available. By reducing the inter-stage variance and enhancing the representational coherence, CFF produces more reliable per-stage anomaly maps, leading to significantly improved fused predictions.

Recent works have begun addressing stage heterogeneity through cross-attention mechanisms [19] and uncertainty-aware fusion [20], yet remain supervised or require large normal sets. Concurrently, few-shot adaptation has evolved toward adaptive embedding design [21] and prompt-based calibration [22], yet none address the joint optimization of alignment and fusion under extreme scarcity.

Our main contributions are as follows:

1. We analyze cross-stage representation inconsistency as a limiting factor in few-shot industrial anomaly detection, particularly under scarce supervision.
2. We propose CFF, a simple yet effective module that aligns multi-stage feature distributions via a symmetric similarity loss, stabilizing fusion without modifying the base architecture.
3. Extensive experiments on MVTec AD and VisA show that CFF consistently improves upon the strong April-GAN baseline, achieving gains of up to +1.6% AUROC and +4.1% AP. Ablation studies confirm that representation alignment is key to its performance.

## 2. Related Work

### 2.1. Vision–Language Models for Anomaly Detection

The emergence of large-scale vision–language models such as CLIP [12] has enabled robust zero-shot anomaly detection by aligning image and text embeddings in a shared semantic space. Normal regions are expected to align well with prompts like “a photo of a normal [object]”, while anomalies yield low similarity [13]. WinCLIP [13] improves localization via class-conditional prompting and sliding window inference. However, CLIP’s features are optimized for generic object recognition—not fine-grained industrial defects—and often lack sensitivity to subtle textures or structural deviations.

Similarly, AnomalyCLIP [23] integrated multi-prompt ensembles to better capture diverse anomaly types. More recent works introduced rectification mechanisms to address CLIP’s limitations in distinguishing subtle abnormal cues.

To bridge this gap, recent methods combine CLIP with self-supervised ViT backbones. April-GAN [16] extracts multi-stage features from CLIP’s ViT encoder and reconstructs normal patch features in a few-shot setting using adversarial prompting. AD-DINOv3 [15] adapts DINOv3 [15], a large-scale self-supervised ViT, by extracting hierarchical tokens and aligning them with CLIP-derived textual prompts via lightweight adapters. To counteract DINOv3’s global bias, it refines the [CLS] token using an Anomaly-Aware Calibration Module (AACM). Final anomaly maps are obtained by computing cross-modal similarities per stage and averaging them. While effective, both approaches fuse stage-wise scores without explicitly reconciling the heterogeneous nature of features across depths—an issue our method resolves through few-shot-guided cross-stage calibration.

Prior to vision–language models, unsupervised anomaly detection relied on modeling normal feature distributions: PaDiM [7] uses multivariate Gaussian statistics; PatchCore [11] builds a memory bank for nearest-neighbor search; and SPADE [24] employs random Fourier features for efficient similarity approximation. Recent works have further optimized background modeling from the perspective of feature constraints and model lightweighting: You et al. [25] proposed a superpixel-guided background inpainting strategy to construct a clean background dictionary and avoid anomaly contamination, while Zhang et al. [26] designed a lightweight CNN based on residual learning and background estimation to reduce the dependence on large-scale normal samples. However, these methods require abundant normal data for reliable estimation—making them ill-suited for few-shot settings. In contrast, vision–language integration injects strong semantic priors with minimal supervision—a regime where our method is designed to excel.

### 2.2. Multi-Stage Feature Fusion

Fusing features across network depths is a well-established strategy to balance local detail and global semantics. In segmentation, U-Net [27] uses skip connections; FPN [28] constructs multi-scale representations via top-down pathways; CBAM [29] applies channel- and spatial-wise attention for adaptive weighting.

In anomaly detection, however, fusion remains largely heuristic. Most methods including April-GAN and AD-DINOv3 adopt *late fusion*: generating independent anomaly maps per stage and combining them via summation or averaging. This implicitly assumes that anomaly scores from different stages are inherently compatible in terms of scale, distribution, and semantic granularity. Yet shallow layers capture high-frequency patterns (e.g., edges and textures) at fine spatial resolution, while deep layers encode coarse structural semantics at low resolution, resulting in complementary but heterogeneous representations. This heterogeneity challenge is also widely verified in hyperspectral anomaly detection, where the fusion of shallow spatial gradient features and deep semantic spectral features faces similar misalignment problems, and existing works have confirmed that local contrast enhancement and explicit feature constraint can effectively alleviate this problem [30,31]. Without explicit reconciliation, naive fusion risks amplifying noise from shallow layers or suppressing meaningful signals from deep ones, especially when supervision is scarce. Recent work such as CATANet [32] demonstrates that content-aware token aggregation can replace naive averaging for more effective multi-stage fusion.

Crucially, unlike segmentation or detection, anomaly detection lacks pixel-level supervision—even in few-shot settings—making learned fusion (e.g., via attention or gating) prone to overfitting or degenerate solutions. As a result, uniform averaging remains common, trading adaptivity for stability. Our approach instead ensures compatibility before fusion: by calibrating features across stages to share consistent semantics and geometry in the anomaly scoring space, simple averaging becomes both effective and robust.

### 2.3. Feature Calibration and Internal Consistency

Feature alignment is central to representation learning. Knowledge distillation [33] matches teacher–student output distributions; self-distillation extends this idea by using the model itself as its own teacher, enabling internal regularization without external supervision [34]. Contrastive learning (e.g., SimCLR [35]) aligns augmented views of the same instance. In few-shot learning, self-calibration modules refine prototypes using unlabeled data [36].

Closer to our goal, internal consistency methods encourage agreement between different parts of a model. For example, MUSC [37] enforces multi-stage similarity consistency under normal conditions, but does so in an unsupervised manner, without leveraging few-shot guidance. Our work differs fundamentally: we enforce cross-stage alignment specifically in the anomaly scoring space, using a symmetric similarity loss where adjacent stages serve as mutual references. Critically, this alignment is guided by few-shot normal samples, enabling the calibrator to learn stage-specific notions of “normality”. This grounding ensures that alignment reflects normal behavior, not just arbitrary coherence—a key insight enabling robust detection even with a single reference image.

Our symmetric similarity loss is inspired by recent advances in cross-domain contrastive alignment [38] and hierarchical consistency modeling through multi-level graph contrastive learning [39], but is uniquely grounded in few-shot normal samples. The lightweight affine adapter follows LoRA [40] and CLIP-Adapter [41], both demonstrating that minimal calibration suffices to bridge semantic gaps in industrial vision, without retraining backbone weights or adding inference latency.

Recent efforts explore hierarchical feature alignment to reconcile semantic gaps across ViT stages [42,43], or calibrate the fusion of vision–language and self-supervised representations using minimal supervision [43].

## 3. Method

### 3.1. Preliminaries: April-GAN

APRIL-GAN [16] extracts multi-stage patch features from a ViT [44] encoder (e.g., at layers *S* = 6, 12, 18, and 24) and maps each stage’s features into the CLIP text embedding space via stage-specific linear projections. Text embeddings are derived from hand-crafted prompts (e.g., “a photo of a normal [object]”), and per-stage anomaly scores are computed via cross-modal similarity. The final anomaly map is obtained by summing scores across stages.

Crucially, APRIL-GAN treats each stage independently: its projection layers are trained without explicit coordination, and no mechanism aligns the semantic or statistical properties of features across depths. As a result, features from different stages—though semantically complementary—exhibit significant heterogeneity in terms of their granularity, scale, and distribution. This limits the effectiveness of naive summation-based fusion, especially under scarce supervision. Our method addresses this gap by introducing few-shot-guided cross-stage calibration to harmonize representations before fusion.

### 3.2. Calibrated Feature Fusion (CFF)

**Figure 1:**
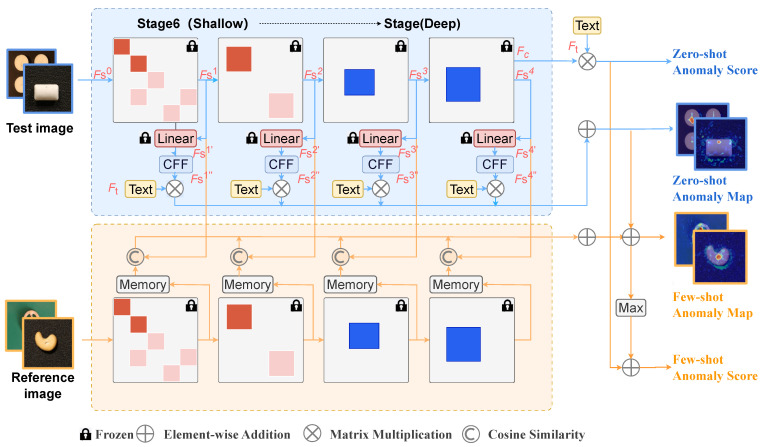
Overview of the proposed Calibrated Feature Fusion (CFF) framework. After independent linear projections, a learnable calibration block $C_{n}$ is applied to align feature distributions across stages. A symmetric similarity loss enforces consistency between adjacent stages, enabling more reliable fusion for anomaly detection.

To address the *heterogeneity across ViT stages*—i.e., the misalignment of feature statistics, granularity, and semantic scale that undermines the reliable fusion of per-stage anomaly scores—we propose Calibrated Feature Fusion (CFF), which introduces a lightweight calibration block $C_{n}$ (defined as a learnable affine function $C_{n}:R^{H\times W\times C}\to R^{H\times W\times C}$) after each linear projection to explicitly align feature distributions across stages. As illustrated in Figure 1, the calibrated feature for stage *n* is

(1) $$F_{n}^{\prime \prime }=C_{n}\left(F_{n}^{\prime }\right)=W_{n}F_{n}^{\prime }+c_{n},$$

where $W_{n}\in R^{C\times C}$ and $c_{n}\in R^{C}$ are additional learnable parameters. Crucially, unlike April-GAN’s independent training, the calibration blocks $\left\{C_{n}\right\}$ are *jointly optimized* with a cross-stage alignment objective, ensuring that $\left\{F_{n}^{\prime \prime }\right\}$ resides in a coherent embedding subspace.

In our implementation, we extract feature maps from four stages (S={6,12,18, and 24}) of the ViT-L-14-336 backbone, where stages 6 and 12 correspond to shallow representations and stages 18 and 24 capture deeper semantics. For each stage n∈S, the calibration parameters $W_{n}$ (scale) and $c_{n}$ (bias) in Equation (2) are initialized as identity scaling ($W_{n}=I$) and zero bias ($c_{n}=0$), ensuring that CFF is *identity initialized* and behaves as a passthrough transformation at initialization. During few-shot inference, calibrated features ${\left\{F_{n}^{\prime \prime }\right\}}_{n\in S}$ are upsampled to the highest resolution (stage 6) and fused via element-wise summation before computing similarity scores against the CLIP text embeddings of “a photo of a normal [object]” and “a photo of an anomalous [object]”.

#### 3.2.1. Alignment Loss

To ensure consistent semantic representations across stages, we minimize a symmetric alignment loss on L2-normalized calibrated features. This loss is a lightweight, task-specific regularization designed to address a practical bottleneck in few-shot anomaly detection: the misalignment of multi-stage ViT features under extreme data scarcity. Unlike unsupervised consistency methods that require large normal datasets or complex memory banks, our alignment objective operates solely on the few available normal reference images, making it strictly label-free, plug-and-play, and deployment-friendly. As empirically validated in Section 4.3, its hierarchical design (local adjacency + global span) yields measurable gains over simpler alternatives, confirming its role as an effective inductive bias for cross-stage calibration.

Specifically, let ${\hat{F}}_{n}^{\prime \prime }=\mathrm{Normalize}\left(F_{n}^{\prime \prime }\right)$ denote the unit norm features. The adjacent-stage loss is

(2) $$L_{\mathrm{adj}}=\sum_{n\in S\setminus \{\max(S)\}}w_{n}\left(1-\mathrm{sim}\left({\hat{F}}_{n}^{\prime \prime },{\hat{F}}_{n+6}^{\prime \prime }\right)\right),$$

where sim(·,·) is the cosine similarity (lowercase, for local adjacency) averaged over spatial locations. Additionally, we enforce global coherence between the shallowest and deepest stages via

(3) $$L_{\mathrm{global}}={∥\mathrm{Sim}\left({\hat{F}}_{\min}^{\prime \prime },{\hat{F}}_{\max}^{\prime \prime }\right)-I∥}_{F}^{2},$$

where Sim(·,·) denotes the cosine similarity (uppercase, for global adjacency) computed between the shallowest and deepest stage features.

The total alignment loss is $L_{\mathrm{align}}=L_{\mathrm{adj}}+\lambda_{g}\cdot L_{\mathrm{global}}$, where $\lambda_{g}=0.1$ balances the global consistency term.

The overall training objective combines pixel-level anomaly segmentation losses with our cross-stage alignment constraint:

(4) $$L_{\mathrm{total}}=\lambda_{f}\cdot L_{\mathrm{focal}}+\lambda_{d}\cdot L_{\mathrm{dice}}+\lambda_{a}\cdot L_{\mathrm{align}},$$

where $L_{\mathrm{focal}}$ and $L_{\mathrm{dice}}$ are the Focal Loss [45] and Dice Loss [46] for precise anomaly region localization, respectively. The weights are set to $\lambda_{f}=0.6$, $\lambda_{d}=0.4$, and $\lambda_{a}=0.1$, chosen empirically to prioritize segmentation accuracy while regularizing feature consistency across stages, which is critical in few-shot settings where overfitting must be avoided.

#### 3.2.2. Two-Stage Training Strategy

Directly training the projection layers $\left\{k_{n},b_{n}\right\}$ and calibration blocks $\left\{C_{n}\right\}$ in an end-to-end manner poses significant challenges. The projection layers are responsible for aligning heterogeneous ViT features into the CLIP embedding space—a task requiring the substantial adaptation of features’ scale and semantics. Simultaneously optimizing the calibration blocks, which aim to further refine these projected features for cross-stage consistency, can lead to gradient conflicts or unstable optimization dynamics, especially under limited few-shot supervision. Moreover, poor initialization of the projections may cause the calibration blocks to adapt to suboptimal or noisy feature distributions, undermining their ability to learn meaningful alignment.

To mitigate these issues, we decouple the learning process into two stages: first establishing a robust base mapping via projection learning, then refining feature coherence through calibration. This sequential strategy ensures that the calibration blocks operate on well-aligned initial features, enabling the stable and effective optimization of cross-stage consistency. We adopt a two-stage training protocol to decouple representation learning from calibration:

1. Stage 1 (Projection Learning): Freeze the ViT backbone and train the initial projectors $\left\{k_{n},b_{n}\right\}$ using standard segmentation losses (Focal and Dice).
2. Stage 2 (Calibration Learning): Freeze $\left\{k_{n},b_{n}\right\}$, initialize $\left\{C_{n}\right\}$, and train them with a combined loss:
  (5) $$L_{\mathrm{total}}=\lambda_{f}\cdot L_{\mathrm{focal}}+\lambda_{d}\cdot L_{\mathrm{dice}}+\lambda_{a}\cdot L_{\mathrm{align}},$$
  where $L_{\mathrm{focal}}$ and $L_{\mathrm{dice}}$ operate on the final fused anomaly map $M=\sum_{n\in S}M_{n}$, and $L_{\mathrm{align}}$ enforces cross-stage consistency.

### 3.3. Inference Protocol

During inference, we strictly adhere to the few-shot protocol of April-GAN [16]: given *K* normal reference images (K∈{1,2,4}), we extract multi-stage ViT features from these references and store them in memory banks. For each test image, we compute two complementary anomaly scores: (1) a *feature-based score* derived from the calibrated multi-stage features via our CFF module, measuring the minimum distance (i.e., 1−maxcosinesimilarity) to the corresponding memory bank entries; (2) a *text-guided score* obtained by comparing the calibrated image features with the anomalous text prompt (“a photo of an anomalous [object]”), following April-GAN’s zero-shot segmentation strategy.

The final anomaly map is formed by fusing these two scores. Crucially, CFF is designed as a strictly additive, plug-and-play module: it operates only on the few-shot calibration path and introduces no changes to the base feature extraction or scoring pipeline. This guarantees that all observed gains stem exclusively from cross-stage alignment, requiring no architectural changes or retraining of the base model.

## 4. Experiments

### 4.1. Experimental Setup

We evaluate on two standard benchmarks in industrial anomaly detection: MVTec AD [2] (15 object categories) and VisA [47] (12 categories). To assess cross-domain generalization, we adopt the *cross-dataset* evaluation protocol from April-GAN: training on one dataset and testing on the other.

The experiments are conducted under few-shot settings. In the few-shot scenario, we follow April-GAN’s protocol with K∈{1,2,4} normal reference images per test category for inference-time adaptation.

We use the CLIP-pretrained ViT-L/14/336 model as the visual backbone, which is identical to April-GAN to ensure a fair comparison. This architecture comprises 24 transformer encoder layers and processes high-resolution inputs (336 × 336), enabling rich multi-scale feature extraction for effective cross-stage fusion.

All models are implemented in PyTorch1.12.1 and trained on an NVIDIA RTX 4060 Ti GPU. We use the Adam optimizer with a learning rate of $1\times 10^{-4}$ and a batch size of 16. Following April-GAN’s dataset-specific strategy, we train for 15 epochs per stage on VisA and 3 epochs per stage on MVTec AD. The random seed is fixed to 42 for reproducibility.

We report comprehensive metrics widely adopted in industrial anomaly detection, covering both pixel-level segmentation and image-level classification. For segmentation, we use AUROC-segm, F1-max-segm, AP-segm, and PRO-segm, and for classification, we use AUROC-cls, F1-max-cls and AP-cls. For more details about the definition of these evaluation metrics, please refer to [2].

### 4.2. Main Results

We present comprehensive cross-dataset few-shot evaluation results in Table 1 (VisA → MVTec AD) and Table 2 (MVTec AD → VisA). CFF consistently improves upon the strong April-GAN baseline across all few-shot settings (1/2/4-shot), both directions, and all metrics, demonstrating robust generalization under domain shift.

The consistent gains across both benchmarks validate that cross-stage representation alignment is a key factor in enhancing multi-stage anomaly detection under limited supervision.

#### Discussion on Metric Trade-Offs

While CFF delivers consistent and substantial gains in pixel-level segmentation metrics (AUROC-segm, AP-segm, PRO-segm, and F1-max-segm) across both cross-dataset directions, we observe a slight decrease in image-level classification metrics (AUROC-cls and AP-cls) in the MVTec AD → VisA setting. This is not an artifact but reflects an inherent design priority: CFF aligns features to maximize spatial consistency for fine-grained anomaly localization, which benefits segmentation at the cost of minor adjustments to global embedding statistics. As shown in Table 2, the largest improvements occur in PRO-segm (+2.3) and AP-segm (+4.1)—metrics directly tied to real-world inspection quality (e.g., detecting small scratches or thin cracks). In contrast, image-level classification serves as a coarse proxy; its modest drop does not undermine its practical utility, especially given that industrial systems typically fuse pixel-level maps with downstream rules or human review. We emphasize that CFF’s core contribution—robust, few-shot-enabled cross-stage alignment—is validated by consistent segmentation gains in *both* directions, with no degradation in any segmentation metric.

### 4.3. Ablation Studies

The proposed Calibrated Feature Fusion (CFF) module integrates feature alignment, similarity modeling, and scoring into a tightly coupled design, where components are mutually dependent and co-adapted during training. Preliminary attempts to ablate individual sub-components (e.g., removing calibration blocks or disabling symmetric similarity loss) led to unstable training or degraded feature representations, yielding non-interpretable results. Therefore, instead of component-wise ablation, we evaluate CFF as a unified module—first by comparing April-GAN with and without CFF, and second by probing the sensitivity of its key hyperparameter $\lambda_{a}$, the weight of the global alignment loss.

As shown in Table 3, introducing CFF yields consistent improvements across all metrics (e.g., +3.0% AP-segm), confirming its effectiveness. Crucially, varying $\lambda_{a}$ from 0.05 to 0.5 induces only minor fluctuations—e.g., AP-segm changes by at most ± 0.25 (57.4–57.9) and AUROC-cls by ≤0.2—all while maintaining substantial gains over the baseline (w/o CFF). This insensitivity to $\lambda_{a}$ further supports our design principle: the alignment mechanism is not a fragile, finely-tuned correction, but a robust structural regularizer that stabilizes cross-stage feature consistency *by construction*. Its role is auxiliary and integrative—reinforcing, not overriding, the core representation learning—which explains both its stability under weight variation and its necessity for end-to-end co-adaptation.

### 4.4. Sensitivity to Shot Number and Calibration Design

To better understand the behavior of Calibrated Feature Fusion (CFF) under varying levels of supervision, we analyze its performance sensitivity across different shot settings (1/2/4-shot) and compare alternative calibration architectures. As shown in Table 1 and Table 2, CFF consistently improves over the April-GAN baseline across all few-shot scenarios on both VisA → MVTec AD and MVTec AD → VisA cross-dataset benchmarks. Notably, the performance gap widens as the number of reference samples increases—e.g., AP-segm gains rise from +2.4% (one-shot) to +3.4% (four-shot) on VisA → MVTec—suggesting that CFF benefits more from richer normal exemplars to learn stable cross-stage alignment.

We further investigate whether the simplicity of our affine calibration block is a key factor in this robustness. To validate the effectiveness and design choices of the proposed Calibrated Feature Fusion (CFF) module, we conduct comprehensive ablation studies on the MVTec AD dataset under four-shot settings. We compare (i) the full model without CFF, (ii) CFF with the default affine calibration block ($F^{\prime \prime }=WF^{\prime }+b$), and (iii) CFF with an alternative MLP-based calibrator. The MLP variant consists of two linear layers with a hidden dimension equal to twice the input feature dimension, and includes BatchNorm1d, ReLU activation, and Dropout (rate = 0.1) in between.

The results in Table 4 demonstrate the following: (1) removing CFF leads to a significant performance drop (e.g., −3.0% AP-segm), confirming its necessity; (2) the simple affine calibrator achieves a better segmentation performance than the more complex MLP variant, while maintaining competitive classification accuracy. This indicates that learnable linear alignment is sufficient for effective cross-stage fusion, and the additional non-linearity and regularization in the MLP do not benefit pixel-level anomaly localization under limited supervision.

Additionally, we evaluate the MLP variant across different shot settings. As shown in Table 5, the segmentation performance consistently improves with more shots, but the affine calibrator maintains a slight edge in AP-segm across all settings, further supporting our design choice.

Moreover, we observe that CFF exhibits minimal variance across random seeds (standard deviation ≈ 0.0 in most metrics), confirming that the symmetric similarity loss stabilizes training even with very limited supervision. This reliability makes CFF particularly suitable for real-world industrial deployment, where consistent performance across product categories and inspection conditions is critical.

This suggests that cross-stage misalignment is primarily a distributional shift (e.g., scale/bias mismatch), which can be corrected by affine transformation without requiring non-linear modeling.

### 4.5. Effect of Calibration Block Design

While our calibration module introduces additional parameters and latency compared to the uncalibrated baseline, it achieves a favorable trade-off between the performance gain and the computational cost. As shown in Table 6, adding the linear calibrator increases inference time from 41.6 ms to 69.6 ms and doubles the parameter count (from 2.25 M to 4.51 M), yet delivers a significant +3.4% AP-segm improvement (see Table 1). Crucially, all variants consume a nearly identical GPU memory (0.0187 GB), indicating no memory overhead during deployment.

In contrast, an MLP-based alternative incurs substantially higher costs (133.8 ms latency, 11.27 M params, and 16.18 G FLOPs), making it impractical for real-time use. This confirms that our lightweight linear design provides effective feature alignment with minimal resource impact, which is suitable for industrial scenarios where model size and memory are critical constraints.

### 4.6. Visualization

**Figure 2:**
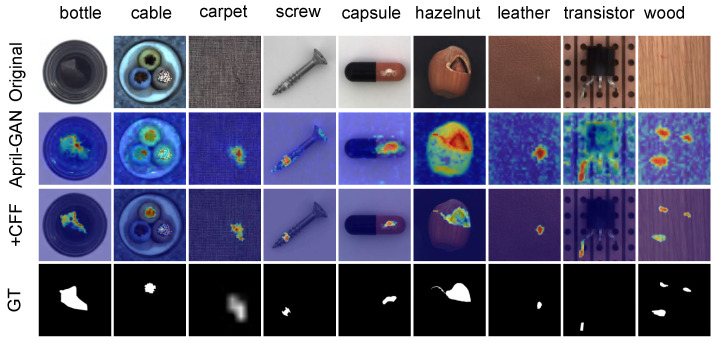
Qualitative results on MVTec AD. Our method (CFF) generates sharper and more accurate anomaly maps compared to April-GAN, especially in fine-grained defects such as scratches on bottles and tiny holes on carpets. For visualization only, all anomaly maps are post-processed with Gaussian filtering and adaptive thresholding; raw maps (unfiltered and unthresholded) are used for all quantitative metrics.

As shown in Figure 2, CFF produces sharper and more accurate anomaly maps, especially in complex textures (bottle) and small defects (carpet and screw), with fewer false positives.

It should be noted that Gaussian filtering and adaptive thresholding are applied only for visual enhancement of the anomaly maps in this figure. These post-processing steps are not used during quantitative evaluation. All metrics (e.g., AUROC and the F1-score) are computed on the raw anomaly maps without any post-processing, ensuring fair and unbiased performance comparison.

## 5. Conclusions

We propose Calibrated Feature Fusion (CFF), a lightweight adapter that enhances few-shot industrial anomaly detection by aligning multi-stage representations. Through a symmetric similarity loss and adaptive training strategy, CFF reduces the inter-stage variance and improves fusion reliability. The experiments on cross-dataset few-shot benchmarks show consistent improvements over April-GAN, with AUROC up to +1.6% and AP up to +4.1%. Future work looks to extending CFF to other backbones and exploring attention-based calibration.

## Figures and Tables

**Table 1:** Performance comparison on VisA → MVTec AD (few-shot only). All values are the mean ± std over three runs. The best results per metric are **bolded**.

Setting	Method	AUROC-Segm	F1-Max-Segm	AP-Segm	PRO-Segm	AUROC-Cls	F1-Max-Cls	AP-Cls
1-shot	SPADE [24]	92.0 ± 0.3	44.7 ± 1.0	–	85.7 ± 1.7	82.9 ± 2.6	89.1 ± 1.0	89.7 ± 1.7
1-shot	PaDiM [7]	91.3 ± 0.7	43.5 ± 1.5	–	78.2 ± 0.8	78.9 ± 3.1	91.2 ± 1.1	91.3 ± 1.2
1-shot	PatchCore [11]	93.2 ± 0.6	53.0 ± 1.7	–	82.3 ± 1.3	86.3 ± 3.0	93.0 ± 1.5	93.8 ± 1.7
1-shot	WinCLIP [13]	**95.3 ± 0.5**	**55.9 ± 2.7**	–	87.1 ± 1.2	93.1 ± 2.3	92.7 ± 1.1	96.5 ± 0.9
1-shot	April-GAN [16]	95.1 ± 0.1	54.2 ± 0.0	51.8 ± 0.1	90.6 ± 0.2	92.0 ± 0.3	92.4 ± 0.2	95.8 ± 0.2
1-shot	Ours (+CFF)	**95.3 ± 0.0**	55.8 ± 0.0	**54.2 ± 0.0**	**91.4 ± 0.0**	**95.2 ± 0.0**	**95.2 ± 0.0**	**97.3 ± 0.0**
2-shot	SPADE [24]	89.2 ± 0.4	42.4 ± 1.0	–	83.9 ± 0.7	81.0 ± 2.0	90.3 ± 0.8	90.6 ± 0.8
2-shot	PaDiM [7]	91.3 ± 0.9	40.2 ± 2.1	–	77.3 ± 2.0	76.6 ± 2.1	88.2 ± 1.1	88.1 ± 1.7
2-shot	PatchCore [11]	92.0 ± 1.0	50.4 ± 1.7	–	78.7 ± 2.0	83.4 ± 3.0	90.5 ± 1.5	92.2 ± 1.5
2-shot	WinCLIP [13]	**96.0 ± 0.3**	**58.8 ± 2.1**	–	**99.4 ± 0.9**	94.3 ± 1.3	94.5 ± 0.8	97.0 ± 0.7
2-shot	April-GAN [16]	95.5 ± 0.0	55.9 ± 0.5	53.4 ± 0.4	91.3 ± 0.1	92.4 ± 0.3	92.6 ± 0.1	96.0 ± 0.2
2-shot	Ours (+CFF)	**96.0 ± 0.0**	57.8 ± 0.0	**55.5 ± 0.0**	92.3 ± 0.0	**94.8 ± 0.0**	**95.3 ± 0.0**	**97.6 ± 0.0**
4-shot	SPADE [24]	92.7 ± 0.3	46.2 ± 1.3	–	87.0 ± 0.5	84.8 ± 2.5	91.5 ± 0.9	90.5 ± 1.2
4-shot	PaDiM [7]	92.6 ± 0.7	46.1 ± 1.8	–	81.3 ± 1.9	80.4 ± 2.5	90.2 ± 1.2	92.5 ± 1.6
4-shot	PatchCore [11]	94.3 ± 0.5	55.9 ± 1.9	–	84.3 ± 1.8	88.5 ± 2.3	92.6 ± 1.6	94.3 ± 1.5
4-shot	WinCLIP [13]	96.2 ± 0.3	59.0 ± 1.8	–	89.0 ± 0.6	95.2 ± 1.6	94.7 ± 0.8	97.5 ± 0.6
4-shot	April-GAN [16]	95.9 ± 0.0	56.9 ± 0.1	54.5 ± 0.2	91.8 ± 0.1	92.8 ± 0.2	92.8 ± 0.1	96.3 ± 0.1
4-shot	Ours (+CFF)	**96.2 ± 0.0**	**59.4 ± 0.0**	**57.9 ± 0.0**	**92.8 ± 0.0**	**96.1 ± 0.0**	**96.1 ± 0.0**	**98.2 ± 0.0**

**Table 2:** Performance comparison on MVTec AD → VisA (few-shot only). All values are the mean ± std over three runs. The best results per metric are **bolded**.

Setting	Method	AUROC-Segm	F1-Max-Segm	AP-Segm	PRO-Segm	AUROC-Cls	F1-Max-Cls	AP-Cls
1-shot	SPADE [24]	95.6 ± 0.4	35.5 ± 2.2	–	84.1 ± 1.6	79.5 ± 4.0	78.7 ± 1.9	82.0 ± 3.3
1-shot	PaDiM [7]	89.9 ± 0.8	17.4 ± 1.7	–	64.3 ± 2.4	62.8 ± 5.4	75.3 ± 1.2	68.3 ± 4.0
1-shot	PatchCore [11]	95.4 ± 0.6	38.0 ± 2.9	–	85.1 ± 2.5	79.9 ± 2.0	81.7 ± 1.6	82.8 ± 2.5
1-shot	WinCLIP [13]	96.1 ± 0.4	**41.9 ± 1.3**	–	80.5 ± 2.3	83.5 ± 4.9	87.1 ± 1.7	85.1 ± 4.0
1-shot	April-GAN [16]	96.0 ± 0.0	38.5 ± 3.7	30.9 ± 0.3	90.0 ± 1.1	**91.7 ± 0.5**	**86.9 ± 0.6**	**93.3 ± 3.3**
1-shot	Ours (+CFF)	**97.3 ± 0.0**	38.9 ± 0.0	**32.1 ± 0.0**	**90.4 ± 0.0**	89.2 ± 0.0	85.2 ± 0.0	90.4 ± 0.0
2-shot	SPADE [24]	96.2 ± 0.4	35.4 ± 0.3	–	85.7 ± 0.1	80.2 ± 8.0	81.7 ± 2.5	82.3 ± 4.8
2-shot	PaDiM [7]	92.0 ± 0.7	21.1 ± 2.9	–	70.1 ± 2.6	67.4 ± 5.1	75.7 ± 1.8	71.6 ± 3.8
2-shot	PatchCore [11]	96.1 ± 0.5	41.0 ± 3.3	–	82.6 ± 2.3	84.6 ± 4.0	82.5 ± 1.8	84.8 ± 3.2
2-shot	WinCLIP [13]	96.8 ± 0.3	43.5 ± 3.9	–	86.2 ± 1.4	81.0 ± 2.4	83.0 ± 1.4	85.8 ± 2.7
2-shot	April-GAN [16]	96.2 ± 0.0	39.3 ± 3.2	31.6 ± 0.3	90.1 ± 0.8	**92.7 ± 3.4**	**87.1 ± 2.3**	**94.2 ± 2.7**
2-shot	Ours (+CFF)	**97.5 ± 0.0**	**42.0 ± 0.0**	**35.2 ± 0.0**	**92.1 ± 0.0**	90.4 ± 0.0	86.6 ± 0.0	91.1 ± 0.0
4-shot	SPADE [24]	96.6 ± 0.3	43.6 ± 0.6	–	87.3 ± 1.1	81.1 ± 0.1	82.7 ± 0.1	83.4 ± 0.3
4-shot	PaDiM [7]	93.2 ± 0.5	24.6 ± 1.8	–	72.6 ± 1.9	72.2 ± 2.9	78.0 ± 1.2	75.6 ± 2.2
4-shot	PatchCore [11]	96.8 ± 0.2	43.9 ± 3.0	–	84.9 ± 1.4	85.3 ± 1.1	84.2 ± 1.3	87.8 ± 2.1
4-shot	WinCLIP [13]	97.2 ± 0.3	47.0 ± 1.1	–	87.6 ± 0.9	87.5 ± 2.1	88.3 ± 1.6	88.5 ± 1.8
4-shot	April-GAN [16]	96.2 ± 0.0	40.0 ± 0.1	32.2 ± 0.1	90.2 ± 0.1	**92.6 ± 0.4**	**88.4 ± 0.5**	**94.5 ± 0.3**
4-shot	Ours (+CFF)	**97.8 ± 0.0**	**44.2 ± 0.0**	**36.3 ± 0.0**	**92.5 ± 0.0**	92.1 ± 0.0	88.1 ± 0.0	92.8 ± 0.0

**Table 3:** Ablation study on MVTec AD (four-shot). Top: main CFF ablation. Bottom: sensitivity to $\lambda_{a}$ (all other settings fixed). The best results per metric are **bolded**.

Method	AUROC-Segm	AP-Segm	AUROC-Cls	AP-Cls
w/o CFF	95.9	54.9	92.5	96.1
w/ CFF	**96.2**	**57.9**	**96.1**	**98.2**
CFF with varying $\lambda_{a}$:				
$\lambda_{a}=0.05$	96.1	57.6	96.0	98.1
$\lambda_{a}=0.10$	**96.2**	**57.9**	**96.1**	**98.2**
$\lambda_{a}=0.20$	96.1	57.7	96.0	98.1
$\lambda_{a}=0.50$	96.0	57.4	95.9	98.0

**Table 4:** Ablation study on CFF: impact of module presence and calibration design (four-shot; MVTec AD). The best results per metric are **bolded**. An upward arrow (↑) indicates that a higher value is preferable.

Setting	Method	AUROC- Segm ↑	AP- Segm ↑	AUROC- Cls ↑	AP- Cls ↑
4-shot	w/o CFF	95.9	54.9	92.5	96.1
	CFF (Affine)	**96.2**	**57.9**	96.1	98.2
	CFF (MLP)	**96.2**	57.4	**96.8**	**98.6**

**Table 5:** Performance of MLP-based calibration across shot settings (MVTec AD). An upward arrow (↑) indicates that a higher value is preferable.

Shot	AUROC-Segm↑	AP-Segm↑	AUROC-Cls↑	AP-Cls↑
1-shot	95.3	52.5	92.8	96.6
2-shot	95.8	56.9	95.7	97.6
4-shot	96.2	57.4	96.8	98.6

**Table 6:** Efficiency comparison of different calibration strategies on ViT-L-14-336 (k=4; bottle category). The linear calibration achieves the best trade-off between performance gain and computational cost.

Metric	No Calibration	Linear Calibration	MLP Calibration
Inference Time (ms)	41.6	69.6	133.8
Throughput (samples/s)	24.05	14.36	7.48
Memory (GB)	0.0187	0.0187	0.0187
Parameters (M)	2.25	4.51	11.27
FLOPs (G)	3.230	6.460	16.183

## Data Availability

The data presented in this study are available in the main article.
